# Taurine supplementation enhances endurance capacity by delaying blood glucose decline during prolonged exercise in rats

**DOI:** 10.1007/s00726-021-03110-8

**Published:** 2022-02-05

**Authors:** Shoichi Komine, Teruo Miyazaki, Keisuke Ishikura, Takashi Matsui, Takashi Miyoshi, Song-Gyu Ra, Akira Honda, Hideaki Soya, Shumpei Miyakawa, Hajime Ohmori

**Affiliations:** 1 Department of Acupuncture and Moxibustion, Faculty of Human Care, Teikyo Heisei University, Toshima-ku, Tokyo, Japan; 2 Faculty of Medicine, University of Tsukuba, Tsukuba, Ibaraki Japan; 3 Joint Research Center, Tokyo Medical University Ibaraki Medical Center, Ami, Ibaraki Japan; 4 Faculty of Management, Josai University, Sakado, Saitama Japan; 5 Faculty of Health and Sport Sciences, University of Tsukuba, 1-1-1, Tennodai, Tsukuba, Ibaraki 305-8574 Japan; 6 Sport Neuroscience Division, Advanced Research Initiative for Human High Performance (ARIHHP), University of Tsukuba, Tsukuba, Ibaraki Japan; 7 Tamagawa Academy, Machida, Tokyo Japan; 8 Institute of Liberal Arts and Sciences, Tokushima University, Tokushima, Tokushima Japan; 9 Department of Gastroenterology and Hepatology, Tokyo Medical University Ibaraki Medical Center, Ami, Ibaraki Japan; 10 Department of Orthopaedic Surgery, Faculty of Medicine, University of Tsukuba, Tsukuba, Ibaraki Japan

**Keywords:** Taurine, Endurance exercise, Lipolysis, Fatty acid oxidation, Gluconeogenesis, Blood glucose concentration, Jugular vein cannulation

## Abstract

Taurine enhances physical performance; however, the underlying mechanism remains unclear. This study examined the effect of taurine on the overtime dynamics of blood glucose concentration (BGC) during endurance exercise in rats. Male F344 rats were subjected to transient treadmill exercise until exhaustion following 3 weeks of taurine supplementation or non-supplementation (TAU and CON groups). Every 10 min during exercise, BGC was measured in blood collected through cannulation of the jugular vein. Gluconeogenesis-, lipolysis-, and fatty acid oxidation-related factors in the plasma, liver, and skeletal muscles were also analyzed after 120-min run. Exercise time to exhaustion was significantly longer with taurine supplementation. BGC in the two groups significantly increased by 40 min and gradually and significantly decreased toward the respective exhaustion point. The decline in BGC from the peak at 40 min was significantly slower in the TAU group. The time when the once-increased BGC regressed to the 0-time level was significantly and positively correlated with exercise time until exhaustion. At the 120-min point, where the difference in BGC between the two groups was most significant, plasma free fatty acid concentration and acetyl-carnitine and *N*-acetyltaurine concentrations in skeletal muscle were significantly higher in the TAU group, whereas glycogen and glucogenic amino acid concentrations and G6Pase activity in the liver were not different between the two groups. Taurine supplementation enhances endurance capacity by delaying the decrease in BGC toward exhaustion through increases of lipolysis in adipose tissues and fatty acid oxidation in skeletal muscles during endurance exercise.

## Background

Glucose is the main energy source during exercise, and maintenance of blood glucose concentration (BGC) is the primary factor affecting endurance exercise performance (Suh et al. 2007). Blood glucose concentration during exercise is constantly maintained by the supply of glycogenolysis and gluconeogenesis in the liver, which are regulated by hormones and nerves (Suh et al. 2007), but it gradually decreases depending on the duration and amount of endurance exercise (Nybo 2003). Indeed, we have shown a significant decrease in BGC during treadmill running to exhaustion in rats (Ishikura et al. 2011). Therefore, preventing a decline in BGC should be an effective strategy for enhancing exercise performance.

Taurine (2-aminoethanesulfonic acid) supplementation has been reported to significantly enhance exercise capacity. Acute ingestion of 1 g taurine before a 3-km running time trial in humans significantly shortened running time (Balshaw et al. 2013). Repetitive ingestion of 6 g/day (2 g three times a day) for 7 days in humans significantly prolonged exhaustion time in incremental cycle ergometer exercise (Zhang et al. 2004). Repetitive ingestion at 20–500 mg/body weight (BW) for 2 weeks in rats prolonged exhaustion time in treadmill running (Miyazaki et al. 2004; Yatabe et al. 2003). Moreover, a significant decrease in exercise capacity was observed in mice with whole-body taurine deficiency due to knockout of the taurine transporter (TAUT) (Ito et al. 2014; Warskulat et al. 2004). Although the mechanisms of the effect of taurine supplementation on exercise performance are not clearly understood, evidence suggests that taurine supplementation at a higher dose, higher frequency, and/or long duration can increase the abundance of taurine in body tissues, including the skeletal muscle and liver, contributing to the enhancement of exercise capacity. In addition, taurine has been suggested to exert this effect by regulating BGC. In a human study, our research group showed that oral taurine administration for 7 days at 6 g/day (2 g per every meal) significantly attenuated the decrease in BGC after 2 h of endurance cycling (Ishikura et al. 2008). However, the same evidence has not been observed in taurine-supplemented rats (Ishikura et al. 2011). This non-significant difference in BGC between the conditions with and without taurine supplementation might be due to the different exhaustion points and different running durations. Thus, we hypothesized whether the BGC at certain points before exhaustion would be higher in taurine-supplemented rats than in non-supplemented controls.

In addition, a previous study suggested that taurine might enhance hepatic gluconeogenesis from amino acids (AAs) during endurance exercise, as the glucogenic AAs, including serine, threonine, and glycine, in the skeletal muscles were significantly decreased in taurine-supplemented rats compared with those in the controls at exhaustion point after exercise (Ishikura et al. 2011). Moreover, taurine has been reported to enhance the blood level of glycerol, which is a substrate of gluconeogenesis due to increased lipolysis in adipose tissues during exercise in humans (De Carvalho et al. 2018). Therefore, we hypothesized that the large differences between the conditions with and without taurine supplementation would be found in these energy metabolic factors, such as gluconeogenesis and lipolysis, at a certain point before exhaustion during endurance exercise.

The present study aimed to evaluate the effects of taurine administration on BGC dynamics under observation over time and on the energy metabolic factors at the effective point of taurine supplementation on BGC.

## Materials and methods

### Animals

Thirty-four male Fischer 344 rats, 8 weeks of age (Japan SLC, Shizuoka, Japan), were used for this experiment, and equally divided into two groups after 1 week of preliminary rearing (3 rats/cage): control (CON) group (*n* = 18) and TAU group (*n* = 16). The taurine supplementation group received 3% taurine solution in distilled water for 3 weeks, whereas the control group received distilled water as a vehicle according to a previously described protocol (Ishikura et al. 2011). After the 3-week administration period, all rats in both groups (BW: CON 233.5 ± 4.1 g, TAU 230.4 ± 3.2 g) were cannulated in the jugular vein. The rats in both groups were further divided into two groups: CON (*n* = 12) and CON120 (*n* = 6) groups, and the TAU (*n* = 10) and TAU120 (*n* = 6) groups, respectively. In the first experiment, rats in the CON and TAU groups were used for the real-time evaluation of BGC during running exercise until exhaustion. In the second experiment, rats in the CON120 and TAU120 groups were subjected to treadmill exercise for 120 min. The exercise time at 120 min was set at the approximate median point in the period (80–150 min), where there were significant differences in BGC between the CON and TAU groups on the real-time blood glucose observation in the first experiment (see Fig. 2).

Animal experiments were approved by the Animal Subjects Committee of the University of Tsukuba, Japan (approval number: 17–076). Rats were provided standard chow and water ad libitum and housed under standard laboratory conditions (20–26 °C, 12-h light–dark cycle).

### Surgery

Cannulation surgery in the CON and TAU groups was performed according to previously described methods (Matsui et al. 2012; Soya et al. 2007). Briefly, the rats were anesthetized by intraperitoneal injection of sodium pentobarbital, and a silicone catheter was inserted into the jugular vein. The inserted catheter was fixed with a silk thread (32 mm), and the external distal end of the catheter was fixed at the nape of the rats. The treadmill exercise experiment was performed 2 days after surgery.

### Treadmill running exercise test

Endurance exercise tests were performed on a treadmill for small animals (FVRO 4E9S-6; Fuji Medical Science Co., Ltd., Chiba, Japan). All rats were familiarized with treadmill running for five consecutive days before the exercise test (Saito and Soya 2004). With this familiarization, the rats ran as much as possible without the electric stimulation equipped with the treadmill machine.

Rats were fasted for 3 h before the exercise at 09:00, and then began running at 12:00. After warming up, the speed of the treadmill was set at 18.8 m/min for 5 min, and then increased to 21.7 m/min (Ishikura et al. 2011). In the CON and TAU groups, cannulated rats were loaded to run until exhaustion, which was judged when the rats could not keep running with electrical stimulation or could not wake up from a lying-down position (Miyazaki et al. 2004; Ishikura et al. 2011). During running, approximately 50 µL of blood was collected every 10 min using a catheter from the jugular vein for glucose measurement. Glucose concentration was measured immediately after blood collection using OneTouch Ultra monitor (LifeScan Japan, Tokyo, Japan). At exhaustion, the rats were euthanized by intravenous injection of sodium pentobarbital (50 mg/kg BW) through the catheter. Furthermore, the rats in the CON120 and TAU120 groups were euthanized by intraperitoneal injection of sodium pentobarbital after treadmill running for 120 min. All rats in the CON120 and TAU120 groups were allowed to run for 120 min, and then the liver and skeletal muscles (soleus, plantaris, and gastrocnemius) were collected, weighed, and immediately stored at − 80 °C until analysis.

### Glycogen content analysis

Glycogen content was measured in the liver and skeletal muscles (soleus and plantaris) in the CON120 and TAU120 groups using the phenol–sulfuric acid method (Lo et al. 1970). Briefly, the tissue was placed in a 30% potassium hydroxide-saturated sodium sulfate solution and heated at 100 °C for 30 min to dissolve the tissue. After mixing several times for complete dissolution of the tissue, the tissue sample was cooled on ice for 15 min, mixed with 95% ethanol, and cooled again on ice for 30 min. Thereafter, the sample was centrifuged at 1700×*g* at 4 °C for 30 min, and the precipitate was dissolved in distilled water to a total volume of 10 mL. This glycogen extract (0.5 mL) with the same volume of 0.5% phenol solution was mixed with concentrated sulfuric acid (2.5 mL). The reaction was incubated at 25 °C for 20 min, and the absorbance was measured at 490 nm. Glycogen content is shown as tissue weight.

### G6Pase activity analysis

G6Pase activity was analyzed according to the previously reported method with modification (Zakko et al. 1998). The liver tissue was homogenized with nine-times volumes of saline on ice and centrifuged at 600×*g* at 4 °C for 10 min. The supernatant was used for analysis of G6Pase activity and total protein content. For analysis of G6Pase activity, the supernatant was diluted 10–20 times with saline and then incubated with 0.1 M sodium cacodylate buffer (*pH* 6.5), 0.1 mL of 10 mM EDTA solution, and 0.2 mL of 50 mM glucose-6-phosphate solution at 37 °C for 15 min. A stop solution (1.0 mL) of 10% trichloroacetic acid was added to the sample, which was then centrifuged for 5 min. Thereafter, the supernatant was mixed with 3.0 mL copper acetate buffer (*pH* 4.0), ammonium molybdate (0.5 mL), and *p*-methylaminophenol sulfate (0.5 mL), and then incubated at room temperature for 10 min. The inorganic phosphorus level was quantified by measuring the absorbance at 880 nm. For analysis of total protein, the supernatant after the first centrifugation was further diluted 20 times with saline, and total protein was measured using a Pierce^®^ BCA Protein Assay Kit (Thermo Fischer Scientific Inc., Rockford, IL). G6Pase activity was expressed as µmol/min/mg protein of liver tissue.

### AA concentration analysis

Free AA concentrations in the liver were measured using an automatic AA analyzer (JLC-300 V; JEOL, Tokyo, Japan) according to previously described methods (Miyazaki et al. 2004; Ishikura et al. 2011; Yatabe et al. 2003). The liver tissue was homogenized with 20-times volumes of 5% trichloroacetic acid solution and centrifuged at 6200×*g* at 4 °C for 30 min. The supernatant was used for AA analysis following four extractions with four-times volumes of diethyl ether. The AA analysis was outsourced to the Research Facility Center for Science and Technology at the University of Tsukuba (Ibaraki, Japan). Amino acids are shown as per tissue weight and were categorized by the energy sources as glucogenic and ketogenic AAs (Namikawa-Kanai et al. 2020). Furthermore, the glucogenic AAs were shown to be pyruvate precursor AAs that would be metabolized to pyruvate (alanine, serine, glycine, threonine, and cysteine), α-ketoglutamate precursor AAs that would be metabolized to pyruvate (glycine, glutamate, arginine, histidine, and proline), succinyl-CoA precursor AAs that are metabolized to pyruvate (valine, isoleucine, methionine, and threonine), and fumarate precursor AAs, which are metabolized to pyruvate (tyrosine and phenylalanine). Ketogenic AAs are metabolized to acetyl-CoA or acetoacetyl-CoA (leucine, isoleucine, lysine, tyrosine, and phenylalanine).

### Analyses of taurine, carnitine, its acetylated forms, and lactate

Taurine, *N*-acetyltaurine (NAT), free carnitine, and acetyl-carnitine (ACT) in the gastrocnemius muscle, as well as lactate in the plasma of the CON120 and TAU120 groups were simultaneously quantified by an HPLC–MS/MS system consisting of a TSQ Vantage triple-stage quadruple mass spectrometer (Thermo Fischer Scientific Inc.) equipped with an HESI-II probe and a Prominence ultra-fast liquid chromatography system (Shimadzu, Kyoto, Japan) according to our previous studies, with modifications (Miyazaki et al. 2017, 2021). In brief, muscle tissue was homogenized with a 10-times volume (*w/v*) of PBS and centrifuged at 3500×*g* at 4 °C for 20 min. Next, 50 µL of the supernatant was mixed with 50 µL of an internal standard (IS) solution containing 100 ng of [^2^H_4_]taurine (C/D/N Isotopes Inc., Quebec, Canada), 1 ng of *N*-acetyl-[^2^H_4_]taurine (synthesized from [^2^H_4_]taurine by reaction with acetate anhydride), 5 ng of l-[^2^H_3_]carnitine HCl (C/D/N Isotopes Inc.), 5 ng of acetyl-l-[^2^H_3_]carnitine HCl (C/D/N Isotopes Inc.), and 500 ng DL-lactate-[^2^H_3_] (C/D/N Isotopes Inc.) in acetonitrile–water (19:1, *v/v*). For lactate analysis, 5 µL of plasma sample was mixed with 50 µL of IS solution. After centrifugation at 2000×*g* for 1 min, the supernatant was evaporated to dryness at 80 °C under a nitrogen stream. The residue was re-dissolved in 100 µL of 0.1% formic acid, and a 5-µL aliquot was injected into the LC–MS/MS system and analyzed in the electrospray ionization (ESI) mode. The general HPLC and MS/MS conditions followed a previously described method (Miyazaki et al. 2021).

Plasma free fatty acid (FFA) concentration was measured using a Wako NEFA C-test kit (FUJIFILM Wako Pure Chemical Corporation, Japan).

### Statistical analysis

Data are displayed as mean ± standard error (mean ± SE). Differences between the two groups and within the same group were analyzed by unpaired Student’s *t *test and repeated-measure ANOVA, followed by Bonferroni *post hoc* test. Correlation relationships were analyzed using Pearson’s correlation coefficients. The significance standard of the confidence rate was set at less than 5% for all statistical analyses. SPSS19 (SPSS Japan, Tokyo, Japan) was used for the statistical analysis.

## Results

### Exercise time and BGC at exhaustion point (the first experiment)

**Fig. 1:**
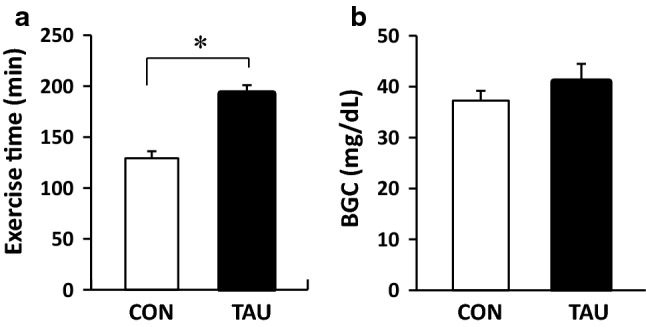
Exercise time until exhaustion (**a**) and BGC at the exhaustion point (**b**). *Abbreviation*: *BGC* blood glucose concentration, *CON* control group, *TAU* Taurine-supplemented group. Values are expressed as the mean ± SE. *P < 0.05 shows significant difference analyzed by unpaired Student’s *t* test

In the treadmill exercise to exhaustion test, rats in the CON group began to drop out as early as 88 min, and the maximum running time was 160 min. In contrast, running times in the TAU group were significantly longer, between 153 and 235 min (Fig. 1a). At the exhaustion point, there was no significant difference in BGC between the two groups (Fig. 1b).

### Over time change in BGC during exercise (the first experiment)

**Fig. 2:**
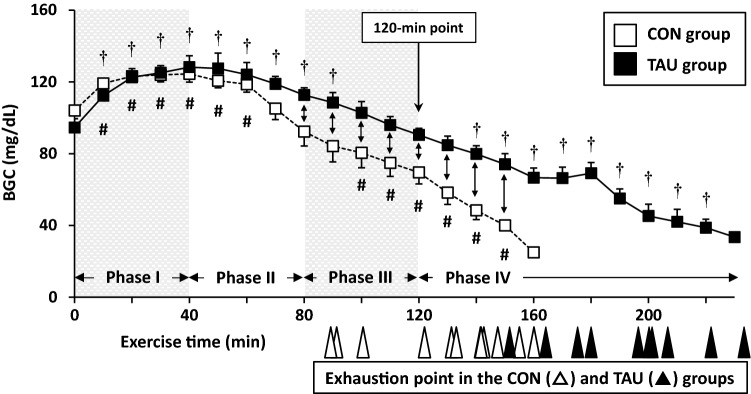
Overtime changes in BGC during treadmill exercise until exhaustion. Blood was collected every 10 min from the jugular vein via cannulation. Exercise period was divided by every 40 min into four phases; Phase I: 0–40 min, Phase II: 50–80 min, Phase III: 90–120 min, Phase IV: over 125 min. Individual exhaustion time is indicated by arrows (white: CON group [*n* = 12], black: TAU group [*n* = 10]) under the X-axis. The 120-min point is the approximate median where there were significant differences in BGC between the two groups at exercise point every 10 min (80–150 min). Values are expressed as the mean ± SE. ^†^*P* < 0.05 and ^#^*P* < 0.05 show significant difference to the respective starting point (0 min) in the TAU and CON groups, respectively, as analyzed by repeated-measure ANOVA followed by Bonferroni *post hoc* test. Arrow with two heads ( ↔) shows a significant difference at *P* < 0.05 between the two groups at each point

In the treadmill exercise, to examine over-time changes in BGC, the exercise period was divided into four phases (Phase I–IV) every 40 min (Fig. 2).

Blood glucose concentration in both groups began to increase significantly immediately after starting the exercise, reached a peak after 40 min (Phase I), and gradually decreased to less than the starting level (0 min point) toward exhaustion (Phase II–IV). In the CON group, BGC was significantly increased from 10 to 60 min compared with that at the starting level and decreased to the starting level after 80 min (Phase II). In the TAU group, the significantly higher BGC than the starting point was maintained for 90 min and regressed to the starting level by 120 min (Phase III). Compared with the respective starting level, BGC was significantly lower between 100 and 150 min in the CON group and between 140 and 220 min in the TAU group. A comparison between the two groups revealed a significant difference in BGC from 80 to 150 min, and the approximate median point in the period was 120 min. Therefore, the running time with a certain difference between the two groups was set at 120 min (the 120-min point) in the second experiment.

### Ratio of BGC change in during exercise and relationship to the exhaustion time (the first experiment)

**Fig. 3:**
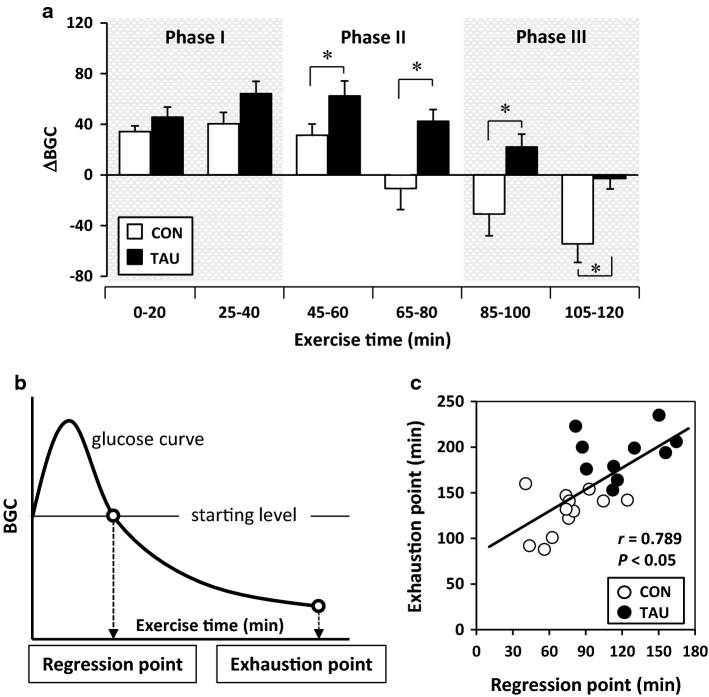
ΔBGC and correlation relationship of regression time to exhaustion time. **a** Overtime change in the ratio of BGC to the starting level (ΔBGC) every 20 min. Values are expressed as the mean ± SE. *P < 0.05 shows a significant difference analyzed by unpaired Student’s *t* test. **b** Scheme of the relationship with BGC progression between regression and exhaustion points in exercise time; the regression point was the exercise time when the increased BGC decreased to the starting level from the peak, and was calculated using the cross-point of the starting level and the regression line that was drawn from the two glucose values before and after crossing the starting level. The exhaust point was the exercise time at exhaustion. **c** Correlation relationship between exhaustion and regression points in exercise time. Correlation was expressed by Pearson’s correlation coefficient

Figure 3a shows the overtime ratio of the change in glucose concentration every 20 min during exercise (ΔBGC). Although there was no difference in Phase I (0–20 and 25–40 min), ΔBGCs in the later periods from 45–60 min to 150–120 min were significantly lower in the CON group than in the TAU group. In the CON group, ΔBGC changed to a negative value from the period to 65–80 min, but it turned to a negative value from to 105–120 min in the TAU group.

The regression points as the exercise time when BGC regressed to the starting level from the peak were calculated from the cross-point of the starting level and regression line that was drawn from the two glucose values before and after crossing the starting level (Fig. 3b). The mean exercise times at the regression point were 75.6 ± 6.9 and 120.1 ± 9.3 min in the CON and TAU groups, respectively (*P* < 0.05). There was a significant positive correlation between the time at the regression point and the exercise time to exhaustion (Fig. 3c).

### Energy metabolic factors in plasma and tissues after 120-min exercise (the second experiment)

**Fig. 4:**
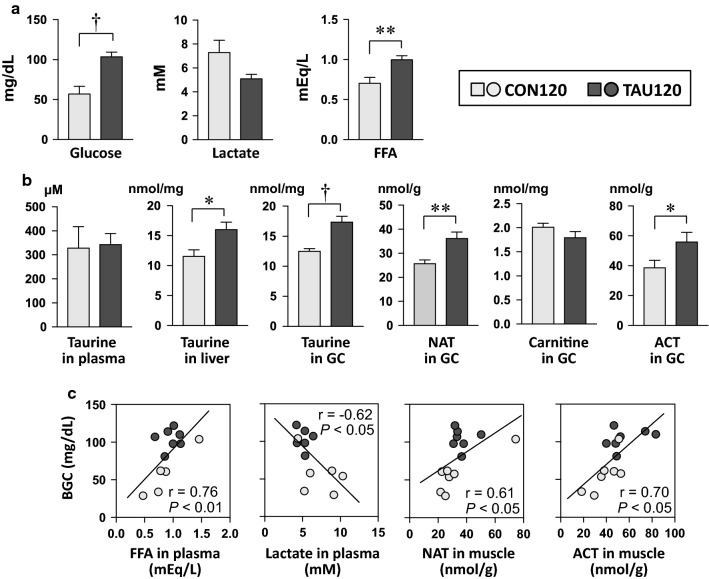
Energy metabolic-related factor concentrations in plasma and tissues after endurance exercise for 120 min. **a** Glucose, lactate, and FFA concentrations in plasma. **b** Taurine, NAT, carnitine, and ACT concentrations in the plasma, liver, and skeletal muscle. **c** Correlation relationships between BGC and these factors, including FFA and lactate in plasma and NAT and ACT in the skeletal muscle. *Abbreviations:* *ACT* acetyl-carnitine, *CON120* Control group at the 120-min point, *FFA* free fatty acid, *GC* gastrocnemius muscle, *NAT* *N*-acetyltaurne, *TAU120* Taurine-supplemented group at the 120-min point. Values in the column graph are expressed as the mean ± SE. *P < 0.05, **P < 0.01, and ^†^*P* < 0.001 shows a significant difference analyzed by unpaired Student’s *t *test. Correlation relationship was analyzed by Pearson’s correlation coefficient

In the 120-min point, plasma glucose and FFA concentrations were significantly higher in the TAU120 group than in the CON120 group (Fig. 4a). In contrast, plasma lactate concentration in the TAU120 group was lower, but not significantly (*P* = 0.06), than that in the CON120 group (Fig. 4a).

Taurine concentration in the liver and skeletal muscle (gastrocnemius) was significantly higher in the TAU120 group than in the CON120 group, but there was no difference in plasma taurine concentration between the two groups (Fig. 4b). In the skeletal muscle, NAT and ACT concentrations in the TAU120 group were significantly higher than those in the CON120 group, but there was no difference in carnitine concentration (Fig. 4b).

There were significant positive relationships in BGC to plasma FFA concentration and NAT and ACT concentrations in the skeletal muscle (Fig. 4c). In addition, plasma lactate concentration was significantly negatively correlated with BGC (Fig. 4c).

### Gluconeogenesis-related factors after 120-min exercise (the second experiment)

**Fig. 5:**
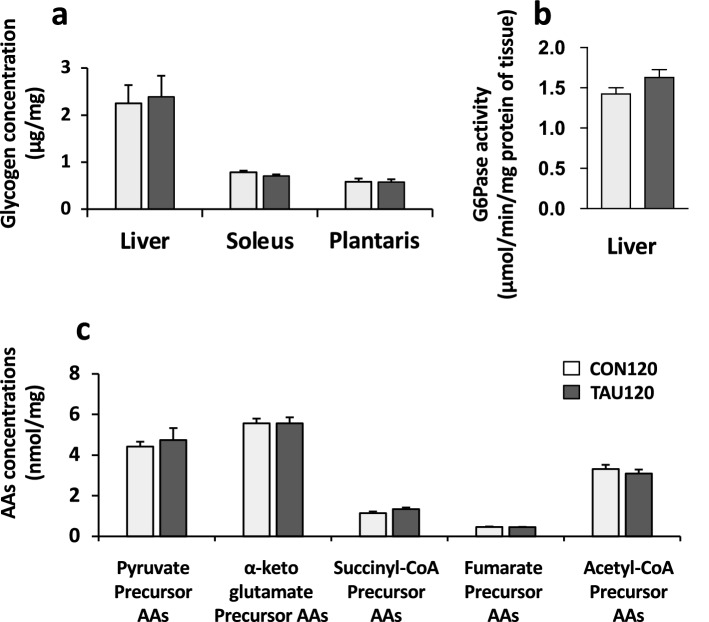
Glycolytic and gluconeogenetic factors after endurance exercise for 120 min. **a** Glycogen content in the liver and skeletal muscles (soleus and plantaris). **b** G6Pase activity in the liver. **c** Glucogenic amino acids (AAs) concentrations in the liver. Pyruvate, α-ketoglutamate, succinyl-CoA, and fumarate precursor AAs: The sum of glucogenic AAs that would be metabolized into pyruvate (alanine, serine, glycine, threonine, and cysteine), α-ketoglutamate (glycine, glutamate, arginine, histidine, and proline), succinyl-CoA (valine, isoleucine, methionine, and threonine), and fumarate (tyrosine and phenylalanine), respectively. Acetyl-CoA precursor AAs: The sum of ketogenic AAs that would be metabolized into acetyl-CoA or acetoacetyl-CoA (leucine, isoleucine, lysine, tyrosine, and phenylalanine). Values are expressed as the mean ± SE

At the 120-min point, glycogen content in the liver and skeletal muscles (soleus and plantaris) was not different between the CON120 and TAU120 groups (Fig. 5a). In the liver, there were also no significant differences in G6Pase activity as well as glucogenic and ketogenic AA concentrations, categorized into the precursors of pyruvate, α-ketoglutamate, succinyl-CoA, fumarate, and acetyl-CoA, between the two groups (Fig. 5b and c).

## Discussion

In the present study, we evaluated the changes in glucose concentration over time during endurance treadmill running in blood samples of rats supplemented with and without taurine. In the overtime observation via cannulation of the jugular vein, BGC dynamics during treadmill exercise, particularly in the declining phase (Phase II–IV) after the once-elevated phase (Phase I), was maintained at a significantly higher level by taurine supplementation. Furthermore, the regression speed of BGC from the peak to the starting level was slower in the TAU group than in the CON group, and there was a significant positive correlation between the regression time of BGC and the exhausted time of exercise. Thus, the efficacy of taurine supplementation in prolonging running time until exhaustion should be mediated by delaying the decrease in once-elevated BGC.

At the 120-min point, the factors related to fatty acid oxidation, FFA concentration in plasma, and ACT and NAT concentrations in the skeletal muscle were significantly higher in taurine-supplemented rats than in the controls. De Carvalho et al. (2018) reported that the increased rate of plasma glycerol concentration, which is a parameter of lipolysis (Jeukendrup et al. 1998), before and after 400 m swimming in maximal effort was significantly higher in swimmers supplemented with taurine (6 g) before 120 min of the exercise than in placebo-treated swimmers. Because FFA is released along with glycerol from triglycerides in adipose tissues to blood during endurance exercise, the significantly higher plasma FFA level in the TAU120 group was likely due to enhanced lipolysis in adipose tissues caused by taurine supplementation during treadmill exercise. Indeed, previous studies have shown that taurine supplementation significantly increases total fat oxidization (~ 16%) after over 90 min and FFA levels in plasma (~ 45%) after 45 min and at exhaustion in submaximal steady-state cycling exercises (Rutherford et al. 2010; Geiss et al. 1994). For this mechanism, taurine has been suggested to augment the action of catecholamines on lipolysis and fat oxidation during exercise (Watt and Spriet 2004; Carvalho et al. 2020) through its direct stimulation of cAMP production via adenylyl cyclase activation, which is involved in the intracellular signal transduction of catecholamines (Chen et al. 2004; Huang et al. 2011).

De Carvalho et al. (2021) recently showed that taurine supplementation (3 g/day) combined with strength and aerobic mixed training for 8 weeks in obese women increased the expression of genes related to mitochondrial activity [such as peroxisome proliferator-activated receptor (PPAR)-γ coactivator (PGC-1α)] and fatty acid oxidation [such as PPARα, PPARγ, lipoprotein lipase (LPL), aconitase 2 (ACO2), hormone-sensitive lipase (HSL), and acyl-CoA oxidase-1 (ACOX1)] in the subcutaneous white adipose tissue (WAT). Furthermore, taurine supplementation alone significantly increased the expression of fatty acid oxidation-related genes (ACO2 and ACOX1) in the tissue. Another study also reported that 5% taurine supplementation in high-fat diet significantly increased the expression of these genes, including PGC-1α, PPARα, PPARγ, and ACOX in the WAT, concomitant with increased body energy expenditure and resting oxygen consumption in obese mice (Tsuboyama-Kasaoka et al. 2006). Because treadmill exercise in rats in the present study was a transience, it was hardly thought that the expression of these genes in the adipose tissues was increased during the exercise. However, the expression of lipolysis-related genes in the adipose tissue might have been upregulated by chronic taurine supplementation for 2 weeks before exercise.

Moreover, ACT and NAT concentrations in the skeletal muscle were significantly higher in taurine-supplemented rats than in the controls at the 120-min point. We have recently shown that ACT and NAT are produced by buffering reactions to the acetyl-moiety of mitochondrial acetyl-CoA derived from fatty acid β-oxidation and acetate, respectively, in the skeletal muscles during endurance exercise (Miyazaki et al. 2021), and are considered as parameters of the oxidation of long- and short-chain fatty acids in the skeletal muscles. Therefore, fatty acid β-oxidation in the skeletal muscles was considered to be increased in taurine-supplemented rats, and this should be upregulated by higher levels of plasma FFA, which is an endogenous PPARα ligand. Taurine might also have a direct promotive effect on fatty acid β-oxidation in the skeletal muscles during endurance exercise, as significant decreases in PPARα target gene expression were found in the taurine-deficient skeletal muscles of TAUT KO mice (Ito et al. 2014). Furthermore, the higher NAT level in the skeletal muscle implied that taurine supplementation increased energy production from acetate in the muscular mitochondria during treadmill exercise. From these results, we suggest that the priority of blood glucose utilization in the peripheral tissues was low after Phase I owing to taurine supplementation. In other words, there is a possibility that lipolysis in the adipose tissue and the utilization of lipids in the skeletal muscles after Phase I was promoted by taurine supplementation, and consequently delayed blood glucose decline in taurine-supplemented rats.

In previous studies, BGC was elevated in the early stage during endurance exercises by sympathetic nerve excitement and the release of hormones, including catecholamines (Williams et al. 1985), glucocorticoids (Drogos et al. 2019), and glucagon (Coker et al. 2000), thereby activating glycogenolysis and gluconeogenesis (Lee-Young et al. 2009). As such, the present study observed a significant increase in BGC in the early stage of exercise, but no effect of taurine supplementation. In the prolonged stage, BGC would decrease along with exercise duration owing to limitations of the maintenance abilities, such as glycogenolysis and gluconeogenesis, in the liver. The present study also showed that at the 120-min point, there were no differences in the factors affecting glycogenolysis and gluconeogenesis between the two groups. Glycogen content in the liver and skeletal muscles was thought to have already reached the depletion level by the 120-min point (Trefts et al. 2015). Both G6Pase activity and glucogenic AA concentrations in the liver, as gluconeogenesis-related factors, were also not different between the two groups at the 120-min point. Indeed, we previously showed that taurine supplementation did not influence the glucogenic AA concentrations in the liver, including serine, threonine, glycine, and alanine, at the exhaustion point of treadmill running in rats (Ishikura et al. 2011). Therefore, taurine supplementation had no effect on glycogenolysis and gluconeogenesis in the declining phase (Phase II–IV), at least at the 120-min point. However, there is a possibility that gluconeogenesis from glycerol might be activated by taurine supplementation during exercise, because increased blood glycerol levels have been reported after taurine supplementation (De Carvalho et al. 2018). Furthermore, taurine may influence renal gluconeogenesis, which is also activated during endurance exercise (Triplitt 2012). These points should be clarified in future studies.

However, the present study did not evaluate the effect of taurine supplementation on the factors regulating BGC at the regression point, as the evaluation of the present study was primarily focused on the 120-min point. Therefore, the mechanism of blood glucose regulation in Phase I needs to be clarified in future studies. Another limitation is that the present study could not measure the sympathetic nervous system and its related hormone levels in the blood. As the volume of blood collected from the jugular vein using cannulation was as small as a few dozen µL, the remaining blood was insufficient for other analyses, such as catecholamine and hormone analyses, after measurement of glucose concentration.

In conclusion, the observation of BCG over time using jugular vein cannulation confirmed that taurine supplementation delayed the decline in once-elevated BGC during endurance exercise. The significantly higher levels of FFA in plasma as well as ACT and NAT in the skeletal muscle at the 120-min point, which showed a significant difference in BGC, suggested that taurine supplementation shifted the priority of energy substrate utilization in the skeletal muscles to fatty acid oxidation during endurance exercise. The consequent sparing effect of taurine on BGC might contribute to enhancing exercise performance.

## Data Availability

Not applicable.
